# Total flavones of *Dracocephalum moldavica* L. protect astrocytes against H_2_O_2_-induced apoptosis through a mitochondria-dependent pathway

**DOI:** 10.1186/s12906-020-2846-4

**Published:** 2020-03-12

**Authors:** Rui-Fang Zheng, Yan-Wen Du, Cheng Zeng, Hui-Fang Wang, Jian-Guo Xing, Ming Xu

**Affiliations:** 1 Xinjiang Medical University, Urumchi, 830000 Xinjiang China; 2 Xinjiang Key Laboratory of Uighur Medicines, Xinjiang Institute of Materia Medica, 140 Xinhua South Road, Urumchi, 830004 Xinjiang China; 3 Department of Clinical Pharmacy, China Pharmaceutical University, 24 Tong jia Lane, P.O. Box 076, Nanjing, 210009 China

## Abstract

**Background:**

The active components of *Dracocephalum moldavica* L. (TFDM) can inhibit myocardial ischemia by inhibiting oxidative stress. However, the effects of TFDM on astrocytes have not been investigated in vitro. The current study aimed to explore whether TFDM protects astrocytes against H_2_O_2_-induced apoptosis through a mitochondria-dependent pathway.

**Methods:**

The human glioma cell line U87 was used to investigate the ability of TFDM to protect astrocytes against H_2_O_2_-induced apoptosis. The cell counting kit-8 assay and flow cytometry were used to detect cell viability, apoptosis, MMP, Ca^2+^ influx and reactive oxygen species (ROS). Lactate dehydrogenase (LDH) and malonic dialdehyde (MDA) levels were measured by ELISA. In addition, protein and mRNA expression changes were detected by Western blotting and qRT-PCR.

**Results:**

TFDM (0.78~200 μg/ml) had limited cytotoxic effects on the viability of U87 cells. Compared with the model group (treated with H2O2 only), cells treated with medium- and high-dose TFDM exhibited reduced MDA concentrations (*P* < 0.05) and ROS production (*P* < 0.05) and decreased MMP (*P* < 0.05) and reduced apoptosis (*P < 0.05*). The percentage of annexin V-FITC-stained cells was markedly suppressed by TFDM, confirming its anti-apoptotic properties. WB results showed that protein expression of Bcl-2-associated X protein (BAX), Caspase-3, Caspase-9, Caspase-12, and B-cell leukemia/lymphoma 2 (Bcl2) was reduced in the TFDM group compared with that in the model group (*P* < 0.05) and that expression of these proteins was normalized by TFDM treatment in a dose-dependent manner. According to RT-qPCR results, TFDM pretreatment resulted in reduced mRNA expression of BAX, Caspase-9, Caspase-12, p38MAPK, and CaMKII and increased mRNA expression of mTOR compared with the model group.

**Conclusions:**

The current study revealed the protective effects of TFDM on U87 cells under oxidative stress conditions through the inhibition of a mitochondria-dependent pathway that is associated with the CaMKII/P38MAPK/ERK1/2 and PI3K/AKT/mTOR pathways.

## Background

Stroke is the second most common cause of death worldwide, and effective treatment methods have not yet been developed [1]. *Dracocephalum moldavica* L., the dry aerial parts of which are widely used as a traditional Chinese medicine in Xinjiang, is a member of the family Lamiaceae [2]. The active components of *D. moldavica* L. (TFDM) have heart- and brain-tonifying functions, activate blood circulation and improve blood stasis [3–6]; therefore, it is mainly used for the treatment of cerebrovascular diseases. TFDM contains the main active components of *D. moldavica* L., accounting for 53.06% of the total activity. The main active ingredients of TFDM are tilianin (13.0 to 17.0%), acacetin-7-O-β-D-glucuronide (10.0 to 13.5%), luteolin-7-O-β-D-glucuronide (8.0 to 9.0%), diosmetin − 7-O- β-D-glucuronide (7.0 to 8.6%) and apigenin-7-O-β-D-glucuronide (2.0 to 3.0%). It has been reported that these flavonoids have anti-inflammatory and antioxidant stress effects [7–12]. In general, flavonoids are the most powerful and widely used bioactive compounds in plants and have cardioprotective and neuroprotective effects and have cardioprotective and neuroprotective effects [13–16]. Single-drug preparations of *D. moldavica* L., called Yixin Badiran Jibuya granules, have been used in the treatment of angina pectoris. Our previous studies found that TFDM can inhibit myocardial ischemia and stroke through the inhibition of oxygen free radical production [17–20], and recent evidence confirms that TFDM functions as an effective and stable free radical scavenger [21]. The purpose of this study was to investigate the role of TFDM in astrocytes, which are the most abundant cells in the central system and comprise approximately 20% of the total brain cells in mammals.

In the brain, neuronal cells are sensitive to oxidative and stress injury, and their survival depends on the protection of neighboring astrocytes against oxidation [22]. It has been reported that neuronal degeneration and astroglial scar destruction caused by astrocyte failure are associated with poststroke inflammatory diffusion and infarct volume in mice [23, 24]. Mitochondria are the main target of oxidative stress injury, and ROS result in an increased free Ca^2+^ concentration in mitochondria, which ultimately leads to apoptosis in astrocytes. Therefore, the survival of astrocytes under oxidative stress may be critical for reducing neuronal death [25]. Human glioma U87 cells have been used as model astrocytes in vitro [26].

Calmodulin kinase II (CaMKII) is an important target for cerebral ischemic nerve injury; phosphorylated CaMKII migrates from the cytoplasm to the cell membrane [27]. The effect is enhanced neuronal discharge and calcium inflow, aggravation of mitochondrial dysfunction and upregulation of the expression of downstream proteins related to apoptosis. The current study explored whether TFDM protects astrocytes against oxidative stress-induced apoptosis by attenuating a CaMKII-dependent mitochondria pathway.

## Methods

### Reagents and antibodies

TFDM was prepared by Xinjiang Institute of Materia Medica (China).

DMEM high-glucose culture medium was obtained from HyClone, and fetal bovine serum was procured from Gibco (USA). Dichlorodihydrofluorescein diacetate (DCFH-DA) was obtained from Sigma. A CCK-8 kit was obtained from Boster (China). We purchased all antibodies from Cell Signaling Technology (USA), Annexin V-FITC and Propidium Iodide Staining Solution were purchased from BD Company (USA), and Mitochondrial Membrane Potential Assay Kit with JC-1 was obtained from Solarbio (China).

### Cell culture and treatment

U87 cells were obtained from the Chinese Academy of Medical Sciences (CAT NO.TCHu38). The cells were incubated in DMEM high-glucose culture medium supplemented with 10% FBS at 37 °C in an atmosphere containing 5% CO_2_.

We established a modified model of H_2_O_2_-induced injury in vitro to simulate pathological changes. U87 cells were incubated with 0.63 mM H_2_O_2_ for 20 min to induce oxidative stress. The cells were divided into three groups: control, H_2_O_2_-induced injury and H_2_O_2_-induced injury pretreated with gradient concentrations of TFDM (100, 50, 25, 12.5, 6.25, 3.13, and 1.56 μg/ml) for 24 h.

### Cell viability assay

U87 cells (1 × 10^4^) were plated in 96-well plates. Six parallel replicates were used for each group. CCK-8 working solution (Boster Biological Technology, USA) was added to each well and incubated for 1.5 h according to the instruction manual. Optical density at 450 nm was detected using a microplate reader (BioTek, USA). Cell viability calculation formula was as follows: [(experimental well-blank well) / (control well-blank well)] × 100%.

### Detection of LDH and MDA

According to the instructions of the LDH and MDA test kits, purchased from Nanjing Jiancheng Company (China), LDH released from cells was measured, and the content of malondialdehyde (MDA) in a cell homogenate was assessed.

### Analysis of apoptosis

Cells were treated as described above; the medium was discarded, and the cells were washed twice with pre-chilled PBS. After trypsin (excluding EDTA) digestion, the cells were collected and washed with pre-chilled PBS. Next, 100 μl of pre-configured 1× buffer was added to resuspend the cells and mixed well; 5 μl of Annexin-V FITC reagent and PI reagent were each added, as was 400 μl of 1 × buffer, and the plates were incubated in the dark. The samples were filtered through a 300-mesh cell sieve, and the apoptotic rate was detected by flow cytometry. The total apoptotic rate was calculated as annexin-V FITC positivity and annexin-V FITC/PI double positivity.

### Evaluation of mitochondrial membrane potential

According to the kit instructions, 1× JC-1 staining buffer was prepared, and 0.5 ml of 1× JC-1 staining buffer was added to each tube. After mixing, the cells were incubated at 37 °C for 30 min and centrifuged at 1000 r • min^− 1^ for 4 min. The cells were collected, and 1 ml of pre-cooled buffer was added and mixed well, followed by centrifugation at 1000 r • min^− 1^ for 3 min; the cells were washed 2–3 times, and 0.5 mL of 1× JC-1 buffer was added and mixed well. The samples were filtered through a 300-mesh cell sieve and examined by flow cytometry. The transition (ratio) of red fluorescence to green fluorescence indicates a decrease in cell membrane potential.

### [Ca^2+^] content assay

Ca^2+^ in U87 cells was detected using Fluo-4/AM (a fluorescent calcium indicator) according to the manufacturer’s instructions. U87 cells were trypsinized and collected according to the above method, and 5 μmol/ Fluo-4/AM was added for 45 min at 37 °C. The cells were washed twice in pre-chilled PBS for 5 min each. The fluorescence intensity of the cells was measured by flow cytometry to represent the content of Ca^2+^.

### Western blot analysis

U87 cell protein was extracted on ice, and a protein lyase inhibitor was added. The protein concentration was determined by the BCA method. Protein loading buffer was added, and the proteins were denatured by boiling at 95 °C for 5 min in a metal bath. After SDS-polyacrylamide gel electrophoresis, the proteins were transferred to a PVDF membrane. After blocking with 5% skimmed milk powder, the primary antibody was added and incubated overnight at 4 °C. Antibodies against GAPDH, phospho-CaMKII (Thr286), phospho-p38MAPK, phospho-p44/42MAPK, phospho-PI3K, phospho-AKT, and downstream related proteins Bax, Bcl-2, Cleaved caspase-3 and Caspase-12 were used. After the blots were washed to remove excess antibody, they were incubated with the corresponding secondary antibody (1:2000) for 2 h. A photoluminescence solution (Millipore) was used to acquire images with a BioRad imaging system, and the image grayscale was analyzed with Image J (National Institutes of Health, USA).

### Reverse transcription and quantitative real-time PCR

Total RNA was extracted from cultured U87 cells using Trizol reagent. RNA was reverse transcribed into cDNA following the instructions of TIANScript cDNA First Strand Synthesis Kit. The Ct value was detected by quantitative real-time PCR in accordance with the instructions of QuantiNovaTMSYBER® Green PCR Kit and the BioRad CFX Manager detection system. GAPDH was used as an endogenous control. Primers were designed and synthesized by Biomed (Table 1).

**Table 1:** RT-qPCR primer sequences

Primer mane	Primer sequence	Primer mane	Primer sequence
CaMKII	F:GCTGCTATTGTTGTTGGTGTGGAG	GAPDH	F:TGCACCACCAACTGCTTAGC
	R:ACAGGTGAAGCTTGAGAGGAAGTG		R:GGCATGGACTGTGGTCATGAG
mTOR	F:GACCGTCCGCCTTCACAGATAC	P38-MAPK	F:CTTCCAGTCGGCGGTCCATG
	R:GCCGCAGTCCGTTCCTTCTC		R:TCAGATCGGCGTCCATCAAGTG
CHOP	F:AACAGAGGTCACACGCACATCC	Bax	F:CCGCACGTCCACGATCAGTC
	R:CGCTCGTTCTCCTGCTCCTTC		R:CATCACTGCCGCTGCCTCTC
Caspase-9	F:AGAAGACCGGAGTGCAATGGATG	Caspase-12	F:ACACGTCTGGCTCTCATCATCTG
	R:TGGCAGGTAGAGGACAGAGACAG		R:TCTGAGGACTGGTGCTCTGGAC

### Measurement of ROS production

Cell plating and pretreatment methods were the same as above. DCFH-DA was diluted and incubated with cells according the manufacturer’s instructions. Levels of 2′, 7′-dichlorofluorescein (DCF) in the cells were detected by flow cytometry at Ex = 485 nm and Em = 535 nm**.**

### Statistical analysis

SPSS 21.0 was used to analyze the data, and the results are all expressed as the mean ± standard deviation. Three independent experiments were performed to ensure the reproducibility of the results. One-way ANOVA was employed for comparison between groups, and Bonferroni correction was applied. A *p* value < 0.05 was considered statistically significant.

## Results

### TFDM protected U87 cells against H_2_O_2_ injury

**Fig. 1:**
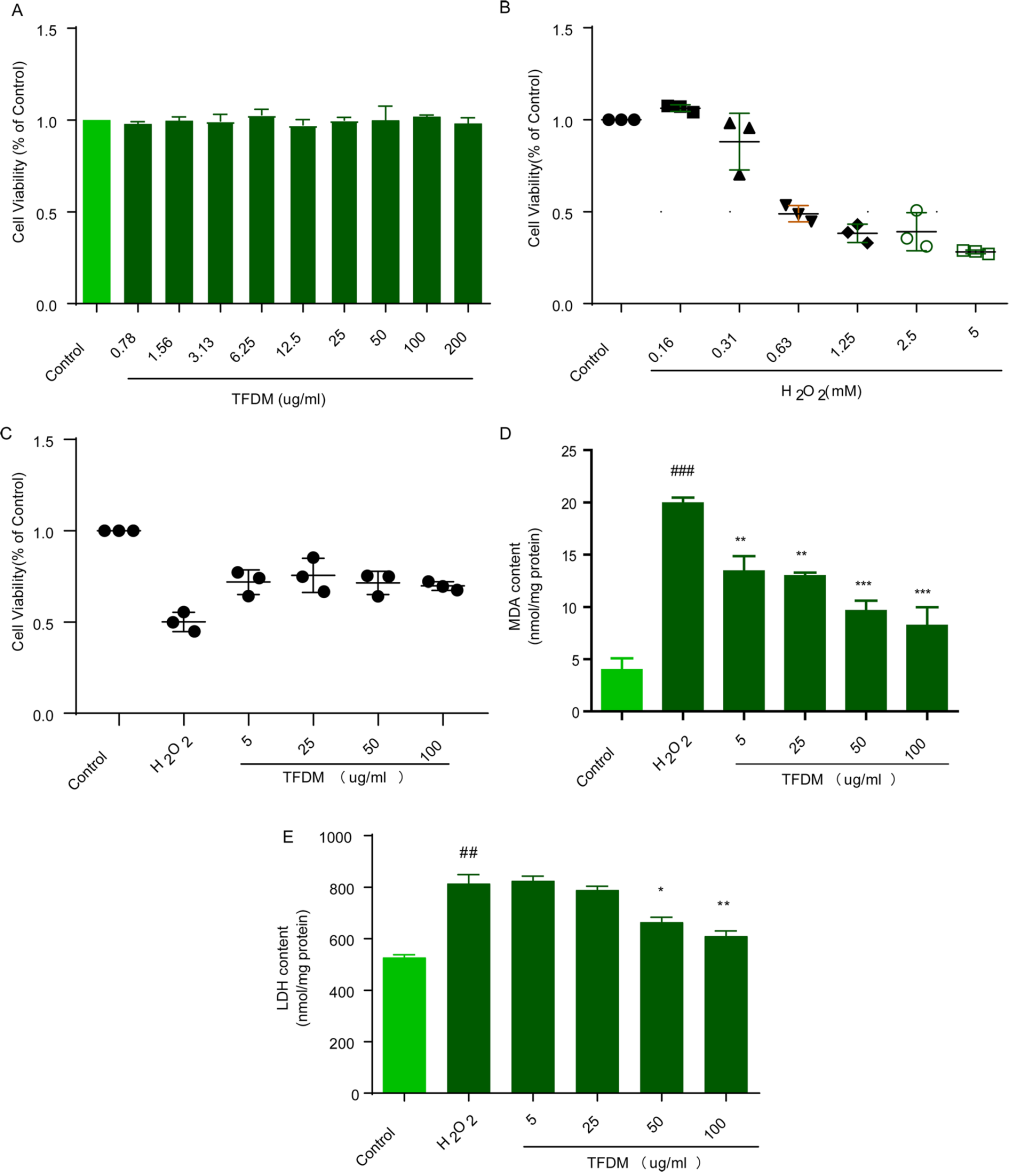
TFDM treatment protected U87 cells against H_2_O_2_-induced cytotoxicity. **a** The cytotoxic potential of TFDM. **b** The effects of different concentrations of H_2_O_2_ on U87 cell viability. **c** TFDM increased cell viability, as evaluated by the MTS assay. **d** TFDM decreased MDA release after H_2_O_2_-induced injury. **e** TFDM decreased LDH release after H_2_O_2_-induced injury. The data are presented as the mean ± SD, *n* = 3. *#p* < 0.05 vs. control; *p < 0.05, **p < 0.01, ***p < 0.001 vs. H_2_O_2_

Based on CCK-8 and LDH kit results, TFDM treatment for 24 h had no significant effect on the viability of U87 cells (Fig. 1a). However, the viability of U87 cell incubated with H_2_O_2_ (0.63–5 mM) for 20 min (Fig. 1b) was significantly decreased. Thus, 0.63 mM H_2_O_2_ was chosen as the dose of H_2_O_2_ for U87 cells. TFDM (5, 25, 50, and 100 μg/ml) dose-dependently restored cell viability (Fig. 1c). Similarly, the level of LDH release and the level of MDA were much higher in the H_2_O_2_ group than in the control group and were dose-dependently reversed by TFDM (5, 25, 50, and 100 μg/ml) (Fig. 1d-e).

### TFDM protected U87 cells against H_2_O_2_-induced apoptosis

**Fig. 2:**
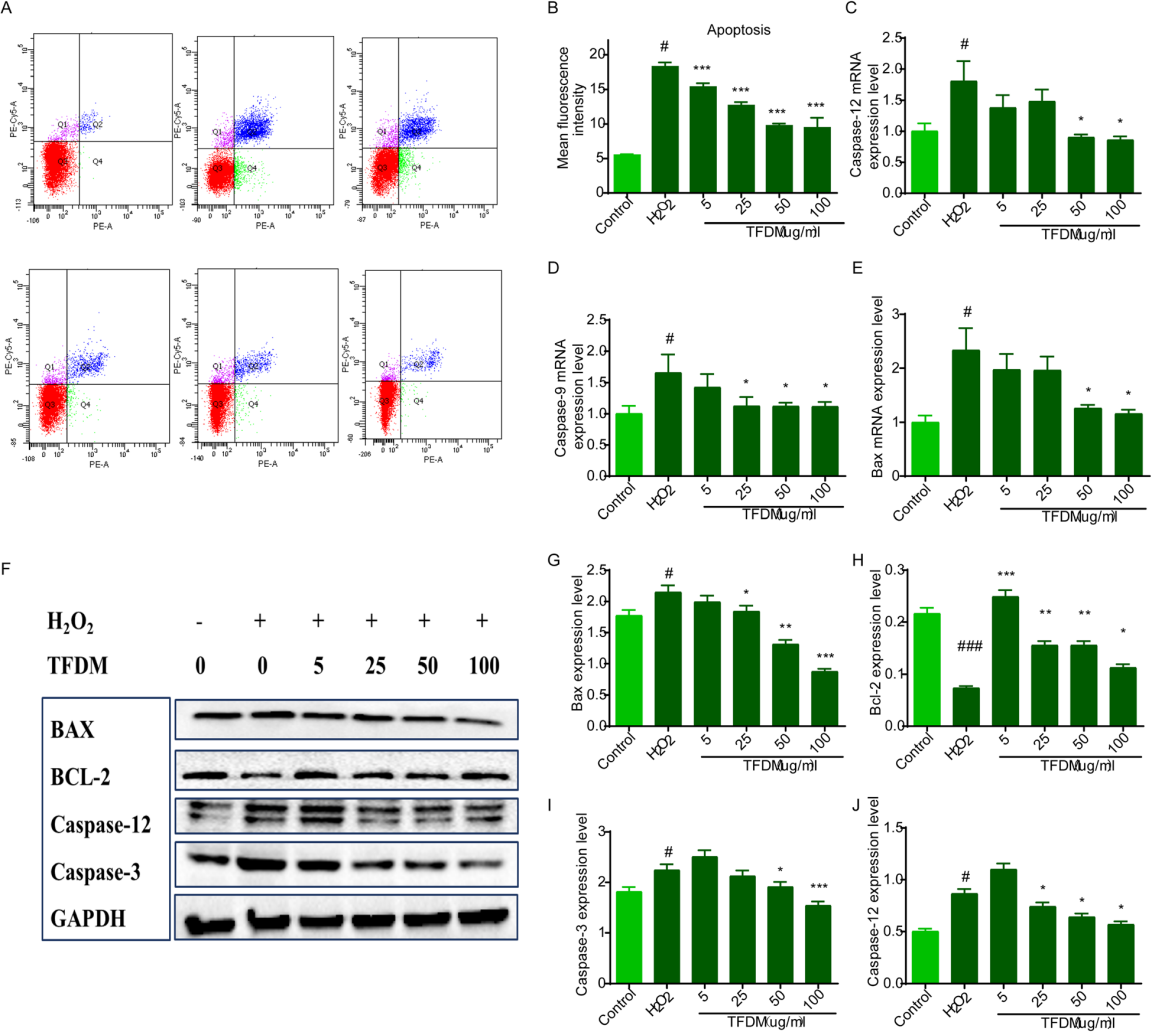
TFDM protected U87 cells against H_2_O_2_-induced apoptosis. **a** Annexin V-FITC/PI double staining was further used to investigate the anti-apoptotic role of TFDM by flow cytometry. **b** TFDM decreased the mean fluorescence intensity of Annexin V-FITC/PI in U87 cells after H2O2-induced injury. **c-e** Representative mRNA levels of Caspase-12, Caspase-9 and Bax, as measured by qPCR, are shown. **f-j** Apoptosis-related protein levels in H2O2-treated U87 cells. The data are presented as the mean ± SD, *n* = 3. *#p* < 0.05 vs. control; *p < 0.05, **p < 0.01, ***p < 0.001 vs. H2O2

H_2_O_2_ treatment enhanced apoptosis from 2.1 to 29.1% (Fig. 2a), whereas TFDM dose-dependently reduced the apoptotic rate (Fig. 2b). Western blotting and qPCR were used to detect apoptotic signal transduction after cell injury, and activation of the mitochondria-related apoptosis proteins Bcl-2, Caspase-3, Caspase-9, and Caspase-12 was observed. Compared with the control group, protein expression of Bax, Caspase-3, and Caspase-12 in the oxidative stress model group increased significantly, and this increase was accompanied by a decrease in Bcl-2. This abnormal expression was reversed by treatment with 5–100 μM TFDM (Fig. 2f-j). qPCR confirmed that the mRNA levels of Bax, Caspase-9 and Caspase-12 were significantly increased and that TFDM pretreatment reduced the level of apoptosis in a dose-dependent manner (Fig. 2c-e). These results were consistent with the Western blotting results. Taken together, the results suggest that TFDM can reverse mitochondrial dysfunction and protect U87 cells against oxidative stress induced by H_2_O_2_.

### TFDM ameliorated mitochondrial damage in H_2_O_2_-injured U87 cells

**Fig. 3:**
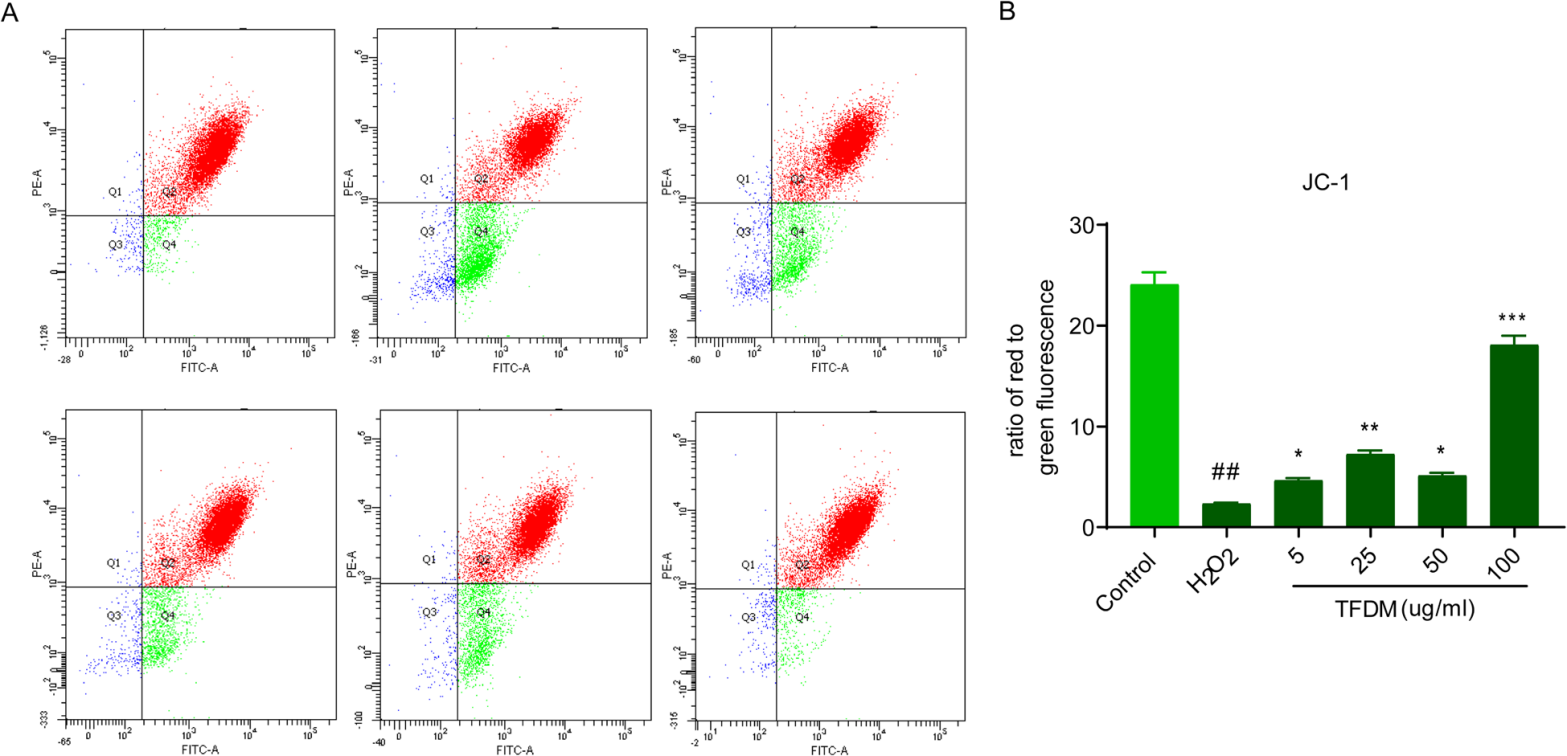
TFDM ameliorated mitochondrial damage in H_2_O_2_-injured U87 cells. **a** Flow cytometry was performed to assess the MMP level in U87 cells treated with H_2_O_2_ and 5, 25, 50 and 100 μg/ml TFDM + H_2_O_2_. **b** The mean fluorescence intensity of images of JC-1 staining. The data are presented as the mean ± SD, *n* = 3. *#p* < 0.05 vs. control; *p < 0.05, **p < 0.01,***p < 0.001 vs. H_2_O_2_

Depolarization of the mitochondrial membrane has crucial roles in stroke and is involved in the mitochondria-mediated apoptosis pathway. It was observed that JC-1 fluorescence at 596 nm (red) and 534 nm (green) increased and that H_2_O_2_ significantly increased MMP in U87 cells (Fig. 3a). However, a dose-dependent recovery in MMP was observed in H_2_O_2_-treated U87 cells treated with gradient concentrations of TFDM (Fig. 3b).

### TFDM inhibited cytosolic Ca^2+^ influx in U87 cells exposed to H_2_O_2_-induced injury

**Fig. 4:**
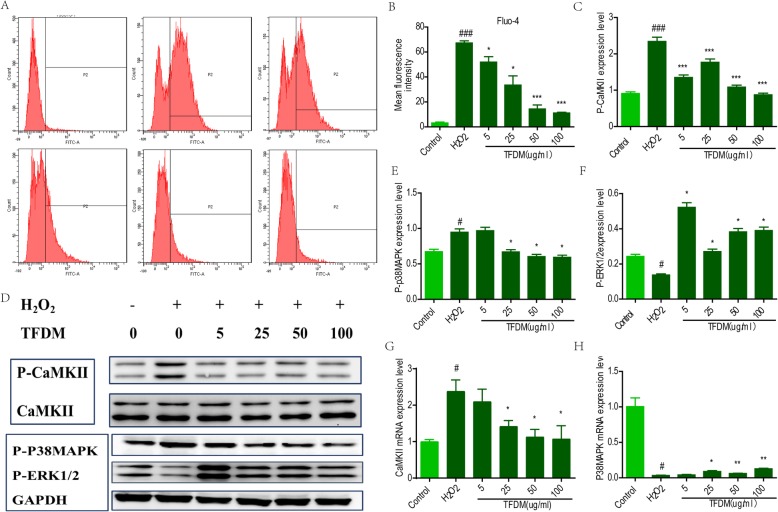
TFDM inhibited the P-CaMKII/P38MAPK/P-ERK signaling pathway. **a** Flow cytometry was performed to assess the Ca2+ level in U87 cells treated with H2O2. **b** The mean fluorescence intensity of images of Fura-4/AM staining. **c-f** Representative Ca2 + −related protein levels of p-CaMKII, p-P38MAPK and p-ERK in H2O2-treated U87 cells. **g-h** Representative mRNA levels of CaMKII and P38MAPK, as measured by qPCR, are shown

It was observed that Fura-4/AM fluorescence was enhanced at 488 nm and 525 nm in the H_2_O_2_ injury group; H_2_O_2_ also promoted Ca^2+^ influx into U87 cells (Fig. 4a). Conversely, treatment with gradient concentrations of TFDM caused a dose-dependent recovery of Ca2+ influx in H_2_O_2_-treated U87 cells (Fig. 4b).

### TFDM inhibited the p-CaMKII/P38MAPK/p-ERK1/2 signaling pathway

To further explore the protective mechanism by which TFDM protects against apoptosis, the possible involvement of CaMKII/P38MAPK/ERK1/2 in astrocytes was examined. We found that phosphorylation of CaMKII, P38MAPK, and ERK1/2 increased dramatically in U87 cells incubated with H_2_O_2_ (Fig. 4c-g). After TFDM exposure, levels of p-CaMKII/p-P38MAPK/p-ERK were decreased. qPCR confirmed that the mRNA expression of CaMKII and P38MAPK was significantly elevated after oxidative damage and that TFDM significantly inhibited mRNA expression of these factors (Fig. 4h-i).

### TFDM ameliorated ROS production in U87 cells exposed to H_2_O_2_-induced injury

**Fig. 5:**
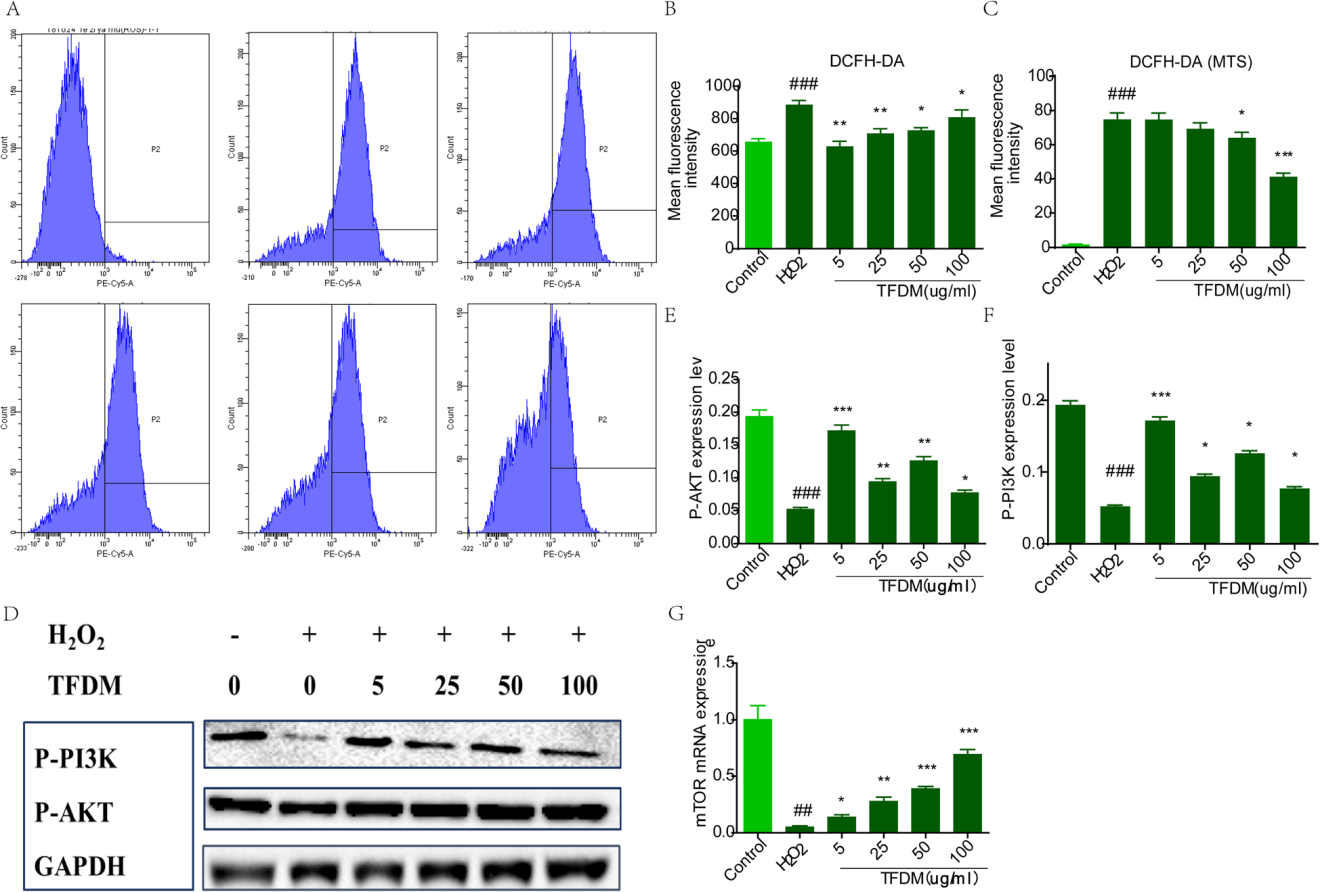
TFDM increased the levels of p-PI3K, p-AKT and mTOR signaling pathway components. **a** Representative images of intracellular ROS stained with DCFH-DA. **b** The mean fluorescence intensity of images of DCFH-DA staining. **c** TFDM inhibited ROS production in U87 cells. Frequency histogram of DCFH-DA, as obtained with a microplate reader. **d-f** Representative ROS-related protein levels of p-PI3K and p-AKT in H2O2-treated U87 cells. **g** Representative mRNA levels of mTOR, as measured by qPCR, are shown

Production of ROS in H_2_O_2_-treated U87 cells was evaluated by DCF with flow cytometry and a microplate reader (Fig. 5a). The results of fluorescence intensity indicated that H_2_O_2_ increased ROS production in U87 cells. After treatment with gradient concentrations of TFDM, ROS levels decreased in a dose-dependent manner (Fig. 5b), which was consistent with the results obtained using the microplate reader (Fig. 5c). As shown in Fig. 5d-f, TFDM reduced levels of p-PI3K and p-AKT in a dose-dependent manner. In addition, TFDM apparently blocked mRNA expression of mTOR in response to H_2_O_2_ (Fig. 5g).

## Discussion

For decades, research on cerebral ischemia has mainly focused on neuronal cells. However, the effect is not obvious [28]. Astrocytes are important for the survival of neurons [21]. One study even suggested that it is more important to maintain and protect the function of astrocytes than neurons in cerebral ischemia [29]. Previous studies on TFDM have mainly focused on myocardial ischemia-reperfusion injury, though effects of TFDM on glial neurons in vitro have not been reported. Therefore, the protective effects of TFDM on astrocytes and the associated mechanisms were investigated in the current study.

After stroke, astrocytes tend to rupture, leading to an imbalance in glutamate absorption and release and an increase in pro-apoptotic factors, which may ultimately accelerate the death of peripheral neurons [30, 31]. Oxidative stress is the basis of the pathogenesis of stroke. Moreover, the survival of astrocytes under oxidative stress conditions may be critical for reducing neuronal death and limiting infarct size [32]. In our study, incubation with H_2_O_2_ caused cellular injury and apoptosis in astrocytes. It has been reported that the active fraction of TFDM, called thistle glycoside, has effective antioxidant activity. In the present study, TFDM increased survival rates and decreased apoptosis rates of astrocytes injured by H_2_O_2._ We first discovered that TFDM had protective effects on glial neurons by inhibiting oxidative stress. The protective effect of TFDM on astrocytes is related to the inhibition of apoptosis. Some antioxidants have been used to treat diseases of the human nervous system [17], which may facilitate the development of TFDM for clinical applications for ischemic injury.

Mitochondria are the source of cellular energy; however, mitochondria are damaged by oxidative stress, resulting in an insufficient ATP supply and excessive accumulation of Ca^2+^ in mitochondria [33, 34] and triggered MPTP opening [29, 35]. Mitochondrial dysfunction leads to the excessive release of mitochondrial apoptosis-related proteins, all of which promote Caspase-dependent neuronal apoptosis [17]. TFDM pretreatment contributes to a significant decrease in MMP in astrocytes. CaMKII was originally identified in the brain, and it is considered a prospective target for cerebral ischemia [36]. Furthermore, phosphorylation of CaMKII activates MAPK family proteins and ERK1/2. We demonstrate that TFDM pretreatment may reduce mitochondrial-mediated apoptosis and levels of p-CaMKII, its downstream MAPKs and p-ERK1/2 in astrocytes.

**Fig. 6:**
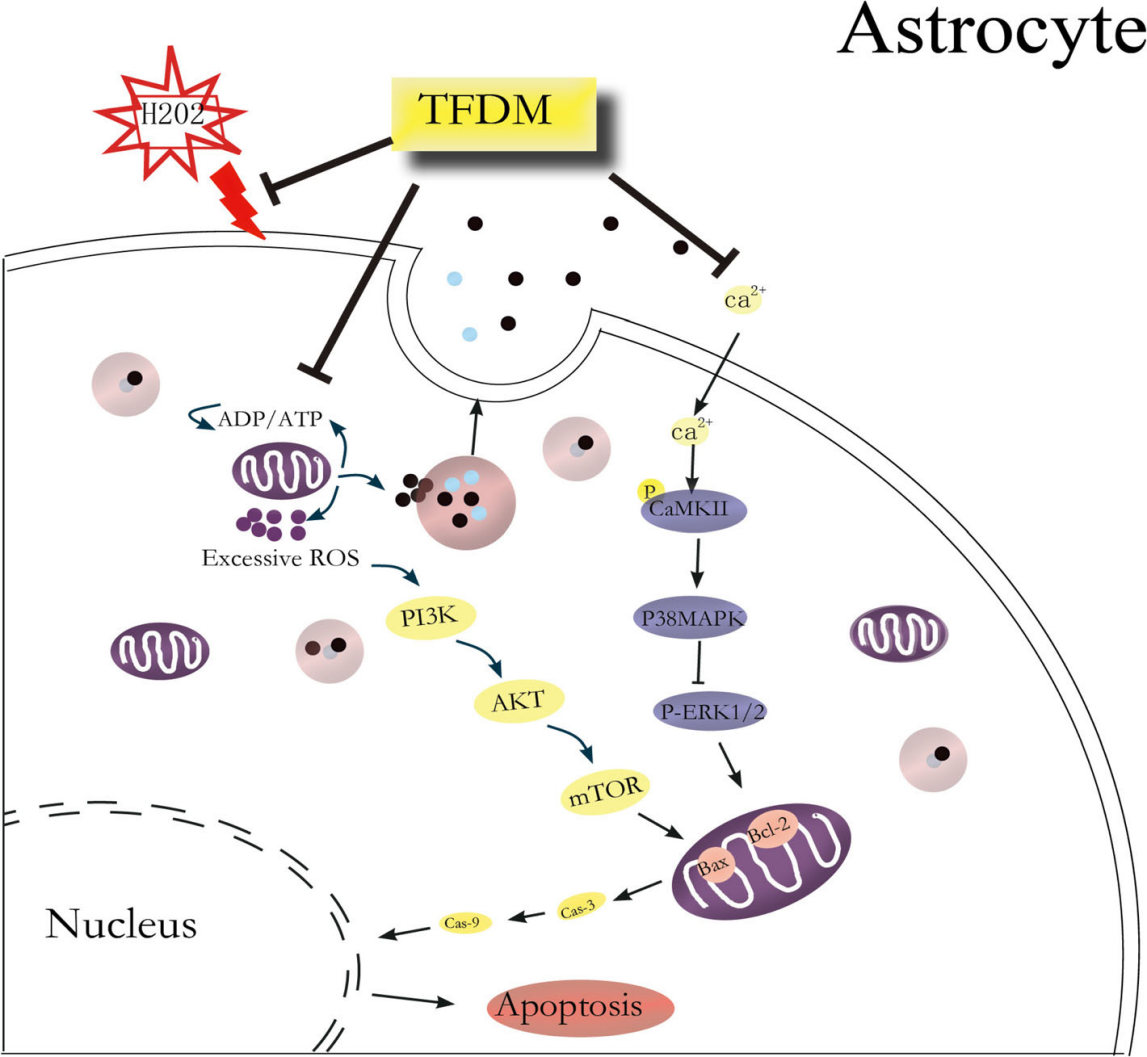
Schematic diagram summarizing the protective effects of TFDM against H_2_O_2_-induced astrocyte apoptosis through a mitochondria-dependent pathway. Proposed protective mechanisms of TFDM against H_2_O_2_ injury via CaMKII-dependent mitochondria-mediated apoptosis and PI3K/AKT/mTOR signaling pathways in astrocytes. H_2_O_2_ induces an increase in [Ca2+], and H_2_O_2_ exposure induces an increase in ROS levels, which might contribute to an elevation of the resting [Ca2+], possibly leading to neurotoxicity in the hippocampus. TFDM relieves CaMKII-dependent mitochondria-mediated apoptosis, including the expression of Bax, Bcl-2, and caspase-3/9 and the activation of p38MAPK and ERK1/2, following cellular H_2_O_2_ injury

After mitochondrial injury, the MPTP opens and releases excessive ROS to induce lipid peroxidation and cytotoxicity, in turn contributing to mitochondrial dysfunction. The present results show that oxidative damage leads to a rapid increase in ROS, phosphorylation of PI3K, AKT and mTOR, and activation of the PI3K/AKT/mTOR pathway, which ultimately promotes apoptosis in astrocytes (Fig. 6).

## Conclusions

In summary, we conducted the first study to show that TFDM protects astrocytes against oxidative stress injury through a mitochondria-dependent pathway. We demonstrate that H_2_O_2_ induces MMP dissipation and consequently leads to mitochondria-dependent apoptosis. TFDM can protect astrocytes against H_2_O_2_-induced apoptosis through a mitochondria-dependent pathway that is associated with inhibition of the p-CaMKII/P38MAPK/p-ERK1/2 signaling pathway and the PI3K/AKT/mTOR pathway.

## Data Availability

The datasets used and analyzed in the current study are available from the corresponding author on reasonable request.

## Abbreviations

BAX: Bcl-2-associated X protein
Bcl2: B-cell leukemia/lymphoma 2
CaMKII: Calcium/calmodulin-dependent protein kinase II
DCFH-DA: 2,7-dichlorodi-hydrofluorescein diacetate
LDH: Lactate dehydrogenase
MDA: Malonic dialdehyde
MMP: Mitochondrial membrane potential
qRT-PCR: Quantitative real-time polymerase chain reaction
ROS: Reactive oxygen species
TFDM: Total flavonoid extract of *Dracocephalum moldavica* L

## References

[1] Donnan GA, Fisher M, Macleod M, Davis SM (2008). Stroke. Lancet.

[2] Liapunova PN, Salo ND, Sergienko TA (1975). Anatomical study of the Moldavian dragon's head herb (Dracocephalum moldavica L). Farmatsiia.

[3] Tan ME, He CH, Jiang W, Zeng C, Yu N, Huang W, Gao ZG, Xing JG (2017). Development of solid lipid nanoparticles containing total flavonoid extract from Dracocephalum moldavica L. and their therapeutic effect against myocardial ischemia-reperfusion injury in rats. Int J Nanomedicine.

[4] Ma L, Zeng C, Zheng R, Jiang W, He C, Xing J (2018). Protective Effects of Tilianin on Brain Tissue in Cerebral Ischemia-reperfusion Injury Model Rats [J]. China Pharm.

[5] Jiang H, Fang J, Xing J, Wang L, Wang Q, Wang Y, Li Z, Liu R (2019). Tilianin mediates neuroprotection against ischemic injury by attenuating CaMKII-dependent mitochondrion-mediated apoptosis and MAPK/NF-κB signaling. Life Sci.

[6] Maimaiti TG, Setiwalidi A (2017). Clinical Drug Use of Vanilla Fragrans in the Treatment of Cardiovascular Diseases in Uygur [J]. Digest World Latest Med Inf.

[7] Zhang N (2019). Insights into the importance of dietary chrysanthemum flower (Chrysanthemum morifolium cv. Hangju)-wolfberry (Lycium barbarum fruit) combination in antioxidant and anti-inflammatory properties. Food Res Int.

[8] Jung HA (2017). Protective effects of flavonoids isolated from Korean milk thistle Cirsium japonicum var. maackii (Maxim.) Matsum on tert-butyl hydroperoxide-induced hepatotoxicity in HepG2 cells. J Ethnopharmacol.

[9] Du Q (2006). Antioxidant constituents in the fruits of Luffa cylindrica (L.) Roem. J Agric Food Chem.

[10] Tian L (2019). Pretreatment with Tilianin improves mitochondrial energy metabolism and oxidative stress in rats with myocardial ischemia/reperfusion injury via AMPK/SIRT1/PGC-1 alpha signaling pathway. J Pharmacol Sci.

[11] Yu N (2015). Simultaneous Determination of Six Active Compounds in Yixin Badiranjibuya Granules, a Traditional Chinese Medicine, by RP-HPLC-UV Method. J Anal Methods Chem.

[12] Qiang L (2015). Study on Pharmacokinetic Characteristics and Metabolic Mechanism of Flavonoids in Pandan. South Med Univ.

[13] Grassi D, Aggio A, Onori L, Croce G, Tiberti S, Ferri C, Ferri L, Desideri G (2018). Tea flavonoids, and nitric oxide-mediated vascular reactivity. J Nutr.

[14] Grassi D, Desideri G, Croce G, Tiberti S, Aggio A, Ferri C (2009). Flavonoids vascular function and cardiovascular protection. Curr Pharm Design.

[15] Vauzour D, Vafeiadou K, Rodriguez-Mateos A, Rendeiro C, Spencer JP (2008). The neuroprotective potential of flavonoids: a multiplicity of effects. Genes Nutr.

[16] Jiang H, Fang J, Xing J, Wang L, Wang Q, Wang Y, Li Z, Liu R (2019). Tilianin mediates neuroprotection against ischemic injury by attenuating CaMKII-dependent mitochondrion-mediated apoptosis and MAPK/NF-kappaB signaling. Life Sci.

[17] Tan ME, He CH, Jiang W, Zeng C, Yu N, Huang W, Gao ZG, Xing JG (2017). Development of solid lipid nanoparticles containing total flavonoid extract from Dracocephalum moldavica L. and their therapeutic effect against myocardial ischemia-reperfusion injury in rats. Int J Nanomedicine.

[18] Zeng C, Jiang W, Yang X, He C, Wang W, Xing J (2018). Pretreatment with Total Flavonoid Extract from Dracocephalum Moldavica L. Attenuates Ischemia Reperfusion-induced Apoptosis. Sci Rep.

[19] Zeng C, Jiang W, Tan M, Xing J, He C (2016). Improved Oral Bioavailability of Total Flavonoids of Dracocephalum moldavica via Composite Phospholipid Liposomes: Preparation, in-vitro Drug Release and Pharmacokinetics in Rats. Pharmacogn Mag.

[20] Jiang J, Yuan X, Wang T, Chen H, Zhao H, Yan X, Wang Z, Sun X, Zheng Q (2014). Antioxidative and cardioprotective effects of total flavonoids extracted from Dracocephalum moldavica L. against acute ischemia/reperfusion-induced myocardial injury in isolated rat heart. Cardiovasc Toxicol.

[21] Fernandez-Fernandez S, Almeida A, Bolanos JP (2012). Antioxidant and bioenergetic coupling between neurons and astrocytes. Biochem J.

[22] Li L, Lundkvist A, Andersson D, Wilhelmsson U, Nagai N, Pardo AC (2008). Protective role of reactive astrocytes in brain ischemia. J Cereb Blood Flow Metab.

[23] Takano T, Oberheim N, Cotrina ML, Nedergaard M (2009). Astrocytes and ischemic injury. Stroke.

[24] Nedergaard M, Dirnagl U (2005). Role of glial cells in cerebral ischemia. Glia.

[25] Poulletier de Gannes F, Haro E, Hurtier A (2011). Effect of exposure to the edge signal on oxidative stress in brain cell models. Radiat Res.

[26] Erondu NE, Kennedy MB (1985). Regional distribution of type II Ca2+/calmodulin-dependent protein kinase in rat brain. J Neurosci.

[27] Xu M, Zhang HL (2011). Death and survival of neuronal and astrocytic cells in ischemic brain injury: a role of autophagy. Acta Pharmacol Sin.

[28] Zhao Y, Rempe DA (2010). Targeting astrocytes for stroke therapy. Neurotherapeutics.

[29] Budd SL, Lipton SA (1998). Calcium tsunamis: do astrocytes transmit cell death messages via gap junctions during ischemia?. Nat Neurosci.

[30] Lin JH, Weigel H, Cotrina ML, Liu S, Bueno E, Hansen AJ, Hansen TW, Goldman S, Nedergaard M (1998). Gap-junction-mediated propagation and amplification of cell injury. Nat Neurosci.

[31] Kim YK, Yoon HH, Lee YD, Youn DY, Ha TJ, Kim HS, Lee JH (2012). Anthocyanin Extracts from Black Soybean (Glycine max L.) Protect Human Glial Cells Against Oxygen-Glucose Deprivation by Promoting Autophagy. Biomol Ther.

[32] Jacobson J, Duchen MR (2004). Interplay between mitochondria and cellular calcium signaling. Mol Cell Biochem.

[33] Nicholls DG (2004). Mitochondrial dysfunction and glutamate excitotoxicity studied in primary neuronal cultures. Curr Mol Med.

[34] Shih EK, Robinson MB (2018). Role of Astrocytic Mitochondria in Limiting Ischemic Brain Injury?. Physiology (Bethesda).

[35] Toussaint F, Charbel C, Allen BG (2016). Vascular CaMKII: heart and brain in your arteries. Am J Phys Cell Physiol.

[36] Nicholls DG, Johnson-Cadwell L, Vesce S, Jekabsons M, Yadava N (2007). Bioenergetics of mitochondria in cultured neurons and their role in glutamate excitotoxicity. J Neurosci Res.

